# Enhancing emotional intelligence and mental well-being for stress management in nursing education – a qualitative study

**DOI:** 10.1186/s12912-025-03338-9

**Published:** 2025-07-01

**Authors:** Annie Haver, Peter Caputi, Kristin Akerjordet

**Affiliations:** 1 NHS-Department of Leadership and Service Innovation, Faculty of Social Sciences, University of Stavanger, Stavanger, Norway; 2 School of Psychology, Faculty of the Arts, Social Sciences & Humanities, University of Wollongong, Wollongong, NSW Australia; 3 SHARE – Centre for Resilience in Healthcare, Faculty of Health Sciences, University of Stavanger, Stavanger, Norway

**Keywords:** Nursing education, Nursing students, Health promotion, Emotional intelligence, Resilience, Stress-management, Mental health/well-being, Higher education-related stress

## Abstract

**Background:**

Nursing students experience high levels of stress due to the combined demands of academic coursework and clinical training. Emotional intelligence (EI) has been shown to support stress management and enhance mental well-being in this context. Despite their potential benefits, there is limited qualitative research on EI-focused interventions in nursing education. The purpose of this study is to explore the impact of a 10-session self-development program on enhancing emotional intelligence and stress management among second-year nursing students in Norway.

**Design:**

An exploratory qualitative design.

**Methods:**

Data were collected from 21 Norwegian university nursing students aged 21–35 using semi-structured interviews. The transcribed interviews were thematically analyzed.

**Results:**

Three main themes were produced: *(1) Fostering a sense of connectedness amongst peers*, including categories such as recognition, emotional safety, trust, common values, and meaningfulness, *(2) Strengthened self-awareness*, including categories such as awareness of one’s own and others’ emotions, honesty, genuineness, upholding a professional image and protecting oneself through emotion regulation, *(3) Development of emotional resilience*, including categories such as adaptability, support from peers, and changing mindset.

**Conclusions:**

This study provides support for the value of self-development programs in a nursing education context. The self-development program created an environment where nursing students felt safe and secure, valued trust, and could communicate openly with each other, which in turn may foster better psychological well-being. Moreover, the findings highlight the value of EI training for nursing students in allowing them to develop awareness of their emotions and those of others, as well as regulate their own emotions to cope better with challenges at university and foster skills that will enable them to deal professionally with future workplace challenges. This supports the value of a stronger emphasis on structured EI and self-development training in nursing curricula, with the potential to inform educational strategies and policy aimed at promoting psychological readiness and long-term professional sustainability.

**Supplementary Information:**

The online version contains supplementary material available at 10.1186/s12912-025-03338-9.

## Background

Research indicates a lack of empirical studies on effective interventions to reduce stress and anxiety in nursing education [1, 2]. Extensive research shows that nursing students experience higher stress levels than their non-nursing peers in higher education [3, 4] due to factors such as clinical performance pressure, academic workload, and emotional demands [1, 3, 5]. Many nursing students are therefore inadequately prepared for the emotional labor these challenges entail [6, 7]. Under such conditions, nursing students may be more prone to maladaptive coping behaviors, which can negatively impact their mental well-being, performance and academic success [8–10]. This is of concern, given that emotional engagement and relational work are central to the nursing role [11–13]. To address these negative outcomes, academic leaders recognize the importance of intervention programs that equip students with resources to effectively manage stress, such as self-care, stress management and resilience training [1, 14, 15]. This need is further underscored by the fact that nursing is globally recognized as a demanding and emotionally taxing profession [16, 17]. Cultivating emotional intelligence (EI) early in nursing education is therefore important to support students’ mental well-being, adaptive capacity, and resilience – better preparing them for future clinical roles [13, 14, 18, 19].

Emotional intelligence (EI) theory posits that EI is crucial for emotion regulation, interpersonal relationships, career development, life satisfaction, and mental health, making it a key factor in personal growth and success [20–23]. EI can be conceptualized as a multifaceted construct, developed as a mental ability [24]. It encompasses the ability to recognize, understand, and manage one’s own emotions and those of others, as well as the capacity to integrate emotional information into cognitive processes. This includes distinguishing between positive and negative emotional influences and using emotional input to guide reasoning and decision-making effectively [25, 26]. Using an EI framework could help illustrate the benefits of understanding and regulating both one’s own and others’ emotions. It serves as a valuable tool to enhance nursing students’ academic and clinical performance, while also reducing the risk of emotional distress [13, 27]. This in turn could contribute to students’ mental well-being, viewed holistically *“as a state of wellbeing enabling people to realize their abilities*,* cope with normal stresses of life*,* work productively and fruitfully*,* and make contributions to their communities”* [28] (p.10).

EI has gained prominence in recent years, underscoring its essential role in preparing nursing students to meet the complex demands of nursing practice. It enables them to transform emotional energy into positive outcomes and navigate challenging situations more effectively [5, 13, 27]. EI also acts as a protective resource, buffering the effects of negative experiences and reducing psychological distress, thereby supporting mental well-being and emotional resilience [12, 29–31]. There is also growing evidence that stress management strategies focusing on self-awareness and the regulation of one’s own and others’ emotions enhance nursing students’ well-being, improve critical thinking and academic performance, and foster stronger patient relationships [27, 32, 33]. EI therefore serves as a vital link between emotional awareness and logical thinking, integrating personal and interpersonal abilities that can be developed over time [34]. By fostering deeper emotional insight, such as self-awareness and empathy, it helps students navigate complex social interactions, make informed decisions, and build meaningful professional relationships [6, 11].

Previous research, including systematic reviews, support the integration of EI into nursing education to strengthen emotional well-being, academic outcomes, and the development of resilience and non-technical skills [6, 14, 19, 30, 35, 36]. While EI has been shown to improve nursing students’ ability to understand and regulate emotions [11, 12, 35, 37], it has yet to be fully recognized or systematically embedded in undergraduate nursing curricula [6, 7, 38]. Identifying effective and sustainable strategies tailored to nursing education is therefore warranted – particularly those that help students to manage maladaptive stressors and prepare for future clinical roles [4, 11, 18]. To address this gap, our study explores the experiences of second-year Bachelor nursing students who participated in a 10-session self-development program designed to enhance emotional intelligence and mental well-being. The aim was to support their ability to manage stress more effectively. This is supported by Xu et al. [5], who suggest that future interventions should prioritize strengthening students’ capacity to apply emotional empathy and EI in practice. Finally, further qualitative research is needed to better understand the role of EI in nursing education and to determine the most effective ways to apply it [14, 39].

## Methods

### Design and setting

This study employed a qualitative exploratory design using a thematic analysis approach. A semi-structured interview guide was used to collect the data. This approach allowed for a flexible, open-ended inquiry into nursing students’ reflections, enabling a rich description of their perspectives without being constrained by predefined theoretical frameworks or hypotheses [40]. The interview guide was validated prior to data collection, and no revisions were deemed necessary (e.g. Vice Dean, nursing students, co-authors). The research was conducted independently and is not part of any larger or ongoing study.

The first author was involved in designing the program in close collaboration with an experienced coach, a registered psychiatric nurse, who facilitated the 10-session program (see Table 1). In addition, the first author conducted the semi-structured interviews. While this dual role may have influenced how participants responded, it also helped build trust and rapport during data collection.

**Table 1:** Contents of the 10-session self-development program (2 h a week)

Sessions	Focus areas	Topics
1	Getting to know each other	Information about the research program, declaration of consent. Getting to know each other, creating trust and curiosity.
2	Self-awareness and self-development	Self-awareness and self-development. Our personality and how it is shaped. Metaphor^a^
3	Role of emotions	Emotions, what are they trying to tell us? Metaphor^a^ Poem^b^
4	Identity and attachment	Identity and attachment: self-criticism, the importance of good and bad relationships. Metaphor^a^
5	Mental well-being	The associations between mental and physical mental well-being. Metaphor^a^
6	Emotional memories	How can emotional memories affect our everyday life? Metaphor^a^
7	Stress management	Stress management: self-care and self-compassion. Metaphor^a^
8	Reason vs. emotions	Reason vs. emotions: Learning how to make wise decisions based on our emotions. Mindfulness can affect the brain. Metaphor^a^
9	Clinical placement	Experiences from clinical placement: How to establish psychological safety in clinical practice situations. Metaphor^a^, Video^c^
10	Life force	How do you find your own life force, personal meaning and joy of life. Metaphor^a^

*a*: See Procedure and Context

*b*: See Procedure and Context. The participants listened to a poem which evoked various emotions

*c*: See Procedure and Context. The participants watched a video from clinical nurse placement before discussing the content further

Participation was voluntary. The participants were second-year Bachelor-level nursing students enrolled at a Norwegian university. According to Lavoie-Tremblay et al. [41], the second-year student nurse cohort is particularly vulnerable to heightened stress levels due to increased clinical performance expectations and limited personal time. The program took place between September 24th, 2019, and December 18th, 2020. At present, the undergraduate nursing curriculum does not include an emotional intelligence (EI) framework in its teaching or learning environments.

The students in this study were completing clinical placements in a range of areas, including psychiatry, medicine and surgery, and home-based care. In Norway, clinical placements account for 50% of nursing education. Our findings may provide insight into whether integrating similar EI-focused content into the formal curriculum could enhance the development of emotional and personal competencies in nursing education.

To situate the study in its broader context, it is noteworthy that on March 12th, 2020, the Norwegian government implemented a nationwide lockdown in response to the COVID-19 pandemic. These restrictions were gradually eased [42] and did not appear to significantly impact the implementation of the study, aside from a few postponements and the introduction of social distancing measures during that period.

### Description of the intervention

The Self-Development Program was not part of the formal curriculum and was conducted independently of any academic course requirements. It took the form of a structured learning initiative that combined guided activities, group discussions and personal reflections, and was designed to enhance participants’ personal and emotional competencies [43–45]. Grounded in research on emotional intelligence (EI) and mental well-being, the program focused on developing key capacities such as self-awareness, emotional regulation, resilience, interpersonal skills, and stress management.

Thirty nursing students participated in the 10-session self-development program conducted in two groups on two separate occasions: (1) late Autumn 2019, and (2) Autumn, 2020. The program consisted of weekly two-hour sessions that aimed to enhance the nursing students’ mental well-being and began with the use of metaphors, poems or videos to promote mutual dialogue and a safe environment. The first session involved providing information about the program and establishing some common rules (e.g. non-disclosure agreement, no mobile phones, mandatory participation etc.). Initially, the nursing students seemed nervous and quiet, but also excited and curious. The coach noticed that the group dynamic gradually improved when participants were encouraged to engage with their peers by using metaphors. Participants were instructed a week in advance to choose a metaphor that encapsulated their self-perception, comparing themselves to a flower or an animal, for example. Subsequently, the participants presented their metaphors and explained why they had chosen them before engaging with their peers in discussions and reflections on how they interpreted each participant’s characteristics, based on their chosen metaphor. Following the ideas of Shared Reading research, the coach read poems aloud with the group. After listening to the poems, all the participants were invited to respond freely. Shared Reading is an established practice originating from the Get into Reading (GiR) program [48]. Shared Reading (reading aloud) interventions have shown promising results in terms of improved emotion regulation, concentration, memory, and mental well-being [46, 47]. In this study, the use of metaphors, along with discussion about film clips and poems, enhanced mutual dialogue and reflections, thus contributing to greater openness and understanding of other world views.

Each session was conducted in a room on campus that provided a suitable atmosphere for group reflection. To add to a sense of conviviality, food and drinks were also served. To facilitate eye contact and strengthen relationships with fellow group members, the participants were placed in a large circle without a table, enhancing a sense of safety and productivity. Once the nursing students had agreed to participate, attendance was mandatory, although absence from two of the ten group sessions was allowed.

### Participants and recruitment

Prior to the study, an open invitation to participate in the self-development program was distributed via the university’s website and social media platforms (e.g. Facebook, Instagram). Approximately 320 s-year undergraduate nursing students were reached. The program was also promoted through announcements on the student learning platform (Canvas) and by the first author in classroom settings. To attract interest, the tag line for the program was: *“How to build psychological muscles?”* Students who were interested signed up using an online questionnaire tool, Microsoft Forms. A “first come, first served” principle was applied, with a cap of 15 participants per group. As previously point out, thirty students participated in the self-development program.

Recruitment for the study was guided by a purposive, criterion-based sampling strategy. Eligible participants were second-year Bachelor’s students enrolled in a Norwegian university who had attended at least eight out of ten sessions of the program. This criterion ensured that participants had sufficient exposure to meaningfully reflect on the experience. Students who had not completed the program or who were absent from several sessions were excluded. The sampling strategy aimed to support the study’s exploratory and in-depth focus, enabling rich insights into students’ lived experiences with the self-development program.

At the end of the 10-sessions, all participants (*N* = 30) were orally invited to the study and were provided with written information and a consent form by the first author. Of these, 21 students met the inclusion criteria and consented to participate in the interview. The average age of participants was 24.6 years (range: 20–36 years). Twenty of the participants identified as women. Additionally, 47.5% reported having at least one year of work experience in healthcare settings (see Table 2).

**Table 2:** Characteristics of the participants (*n* = 21)

Stakeholder group	Age	Previous work experience	Gender
Program I Nursing students (*n* = 10)	21–36 years	1 year as health worker	15 females
Program II Nursing students (*n* = 11)	20–24 years	1.3 years as health worker	14 females, 1 male

### Ethical considerations

The research protocol was approved and accepted by the Norwegian Social Science Data (NSD) (Ethical approval number 2019/692043) and the Vice-Dean of Education at the participating University prior to data collection. According to national regulations, approval from the Regional Committees for Medical and Health Research Ethics was not necessary. Participation was based on informed, voluntary, and written consent. In addition, participants were informed about the right to withdraw from the study at any point without any consequences.

### Data collection

The individual semi-structured interviews were conducted by the first author (AH), either in person on the university campus or via Zoom, a digital communication platform. Zoom sessions were digitally recorded. The interviews lasted approximately 60 min. The interview guide was developed based on the emotional intelligence (EI) research literature and its relation to mental well-being, aimed at gaining an in-depth understanding of nursing students’ experiences in the self-development program (see supplemental file). The interview guide was structured around the following themes: [1] Mental Well-Being (e.g. well-being, closeness to others, positive outlook on life etc.) [2], Emotional Intelligence (e.g. self-reflection, self-awareness, personal and emotional development, emotional regulation etc.). The interview guide was inspired by Wong and Laws’ conceptualization of EI [33]. Questions asked included, for example: (a) In what ways, if any, do you believe that being part of the self-development group has contributed to your self-awareness and personal growth, and (b) In what ways, if any, has participating in the group influenced your ability to regulate emotions, such as stress or frustration, in study situations or clinical practice? (see supplemental file). However, to give the participants an opportunity to familiarize themselves with the study, sample themes and questions were sent about a week prior to the interviews. Beyond this, the interview guide allowed participants to share spontaneous reflections at the end of each interview.

Data saturation was assessed continuously throughout the research process. In line with Thagaard [48], saturation was considered reached when no new insights or themes emerged from the interviews. After the eighteenth interview, the research team observed that the data had become repetitive. Although additional interviews were unlikely to contribute further understanding, all twenty-one participants were included to ensure sample adequacy and rich participant narratives. The final sample was therefore deemed sufficient to support the study’s aim.

### Data analysis

Interviews were transcribed verbatim by an external professional and analyzed inductively. The analysis strategy was based on Braun and Clark’s [49] thematic analysis and included the following six phases: [1] becoming familiar with the data [2], generating initial codes [3], searching for themes [4], reviewing themes [5], defining and naming themes, and [6] producing the report. First, two authors (AH, KA) independently read through the transcribed interviews multiple times while adding comments and notes to gain an overall understanding of the content. Initial ideas were discussed across the dataset. Second, the first author (AH) generated initial codes using the qualitative analysis software program NVivo 12. Third, the first and last authors subsequently sorted the coded data into potential recurring themes covering the nursing students’ experiences and perceptions from the self-development programs. Fourth and fifth, all authors discussed and reached consensus by reviewing, modifying, and making final refinements to the three main themes. Sixth, the first author provided the final report, which was approved by all authors holding PhDs with backgrounds in academic and professional nursing, emotion regulation, emotional intelligence, mental health, and psychology.

## Results

Three main themes were produced from the data analysis: *(1) Fostering a sense of connectedness amongst peers*, including categories such as recognition, emotional safety, trust, common values, and meaningfulness, *(2) Strengthened self-awareness*, including categories such as awareness of own and others’ emotions, honesty, and genuineness, as well as categories related to upholding a professional image and protecting oneself through emotion regulation, and *(3) Development of emotional resilience*, including categories such as adaptability, support from peers, and changing mindset. These themes will now be further elaborated and discussed (see Table 3).

**Table 3:** The three identified themes and categories

Themes	Categories
Fostering a sense of connectedness amongst peers	Recognition, emotional safety, trust, common values, and meaningfulness
Strengthened self-awareness	Awareness of own and others’ emotions, honesty, and genuineness, upholding a professional image and protecting oneself through emotion regulation
Development of emotional resilience	Adaptability, support from peers, changing mindset

### Theme 1: fostering a sense of connectedness amongst peers

Most participants acknowledged the intrinsic value of interpersonal connections, empathizing with the critical role of recognition from their peers [50–52]. They experienced a profound sense of genuinely being noticed and seen:

Even after just a few meetings I noticed there was a good sense of community where everyone looked after one another. I think everyone’s treated each other with respect and been open to what we had to say. We’ve shown a lot of empathy and sympathy for one another. That’s been really reassuring and good. (Nursing Student 11)

We’ve got to know each other in a deeper sense, I see them in the corridors and seeing a face I feel I recognize has meant a lot to me. That feels reassuring and good if I go to the canteen or lecture theatre, then I know who to sit next to. (Nursing Student 14)

For several participants, connectedness played a pivotal role in mitigating feelings of loneliness. This was often related to emotional support and the richness of shared experiences:

There was a very special atmosphere of trust. My problems have got better since I participated because I got to talk about them a little with others who understood. I feel I’m part of something bigger now, and it’s been nice to have this group to turn to, if I needed to talk about something in practice or at school. (Nursing Student 7)

This is the first time I’ve felt I was part of something here at university. I got to meet my fellow students, hear how things are for them and talk a little about how it is for me. I was seen and heard for the first time, something that contributed to my feeling more comfortable. (Nursing Student 17)

Trust is a crucial element of connectedness, with many participants underscoring the importance of being able to share thoughts and feelings in an environment free from judgement or retaliation (no teachers), enhancing emotional safety:

I feel like I’ve got belonging to a group and there’s security in that – of being able to open-up about your own experiences. There’s no other situation where I do that otherwise, so this has been very meaningful. (Nursing Student 21)

The most important thing for me is community, openness, and the support. Also, that I recognize myself in the others, especially when it comes to practice. We got to share experiences and feelings. Everyone could say what was on their mind and nobody judged anyone. (Nursing Student 19)

Most participants also highlighted the significance of shared common values and objectives, particularly in the context of ‘nursing practice’. They also emphasized the importance of receiving constructive feedback from peers:

The fact it was only nursing students was really important because we face the same challenges. When I talk to my husband, who’s an engineer, we talk two different languages. It’s been reassuring and good that we were all nursing students – then we’re on the same path even if we’ve been totally different. (Nursing Student 14)

After presenting my metaphor (e.g. how I perceive myself) I felt completely freed up and that I could speak without a filter. Then I’d opened up. Having someone who understood me and being acknowledged afterwards felt very good. (Nursing Student 10)

In addition, it appeared to be meaningful and exciting for the nursing students to learn more about their peers’ inner thoughts and reflections:

It was very exciting and worthwhile to get to know people (e.g. fellow students) on a deeper level, hearing what they had to say has been both meaningful and educational. I’ve never been part of a group like this before, and it’s been fantastic to hear others’ stories and understand a person’s background. (Nursing Student 4)

The findings suggest that the program fostered a sense of belonging through shared experiences, trust, and emotional safety [45]. Feeling seen and understood by peers reduced loneliness and strengthened interpersonal connections, creating a supportive environment that encouraged openness, reflection, and a deeper understanding of self and others [26, 45, 50, 52].

### Theme 2: strengthened self-awareness

Several aspects of the program focused on self-awareness (acknowledged as a meta-ability of EI), a fundamental aspect/component of personal development and growth [25, 53, 54]. Most participants emphasized the importance of engaging in enhanced inner reflection, which involves deep introspection and mindfulness, examining their core beliefs and behaviors, and mirroring their emotions. Additionally, they recognized the significance of understanding the perspectives of those around them, including patients:

I have been much more reflective around difficult situations. I’ve been quite bad at that because I felt I didn’t want to trouble others about what I found difficult, especially concerning practice and studies. Now I’ve learnt more about myself and others and gained fresh perspectives on how I can become a better person. (Nursing Student 4)

I’ve really learnt a lot about how emotions control us, especially me – and how much easier things are when I become more aware of what kind of emotion I’m experiencing. It’s better to just put words to things – better to let it out than keep it inside. I also think about who I am as a wife, mother, future nurse, friend, and study companion. I’m all these aspects in the relations I have around me. (Nursing Student 14)

Honesty is considered a vital component of self-awareness. Most participants highlighted the value of being honest, particularly with themselves, and acknowledging personal strengths and weaknesses, emotions, and behaviors without resorting to self-deception or denial. By embracing honesty, they were able to gain a deeper understanding of themselves, leading to increased confidence and improved performance in clinical placement:

I’ve got a better understanding of myself, something that can make me a better nurse in the future. I have the courage to stand up for myself and say no to things. I have the courage to refuse things and set boundaries more, both for myself and others, and that has made me more self-confident in practice. (Nursing Student 21)

I feel I create a better and more secure relationship with the patient now and I think as a patient myself I would appreciate it if a nurse was honest and told me they didn’t know what to say because it was really hard. (Nursing Student 4)

A small minority expressed a desire to share more about themselves, but at the same time felt they had to slightly hold back from sharing to prioritize their peers’ needs:

From time to time, I felt I wanted to share, but I thought that perhaps someone had another story to tell that was more important. It wasn’t the group’s fault. I just felt I held back a little because I thought the others needed it more than I did. (Nursing Student 12)

The importance of being genuine, present, and maintaining professionalism with patients was also emphasized:

Now I try to be most spontaneous in my emotions because that’s the most authentic way – being able to support someone well and be authentic but not steal the show. Being a good nurse is about being present and being yourself. (Nursing Student 8)

It’s important to be honest with the patient, but at the same time it feels inappropriate to go to a patient and say I’ve had a bad day. We don’t do that. Every patient is an individual, so even if we should be authentic, we also have to be professional. (Nursing Student 18)

Several participants were nevertheless aware that they sometimes tended to fake and suppress their inner emotions to uphold an image of a professional nursing role:

There are situations where I get angry, annoyed or sad, but I can’t show that to the patient because it can make the situation worse. I have something I call my work-brain – it’s something I screw on when I’m at work. It’s a survival mechanism but it’s for the patient’s own good that I do it. They should feel seen and heard. (Nursing Student 10)

However, several participants emphasized the significance of fostering trustworthy relationships with their nurse mentor, specifically mentioning that a positive relationship with the nurse mentor appeared to reduce their tendency to fake and suppress their emotions:

That thing about faking – I did that a lot in my last practice period, when I wasn’t happy. I always put on a façade then. I didn’t tell the nurse mentor when I felt like that, but she noticed it about me. So, I cried for a bit in the cupboard and then things got a little better afterwards. The nurse mentor saw me and handled it, something that really helped me. (Nursing Student 9)

While some participants acknowledged their vulnerability in being assessed by a nurse mentor they were still able to maintain authenticity when interacting with their patients.

To a large degree I feel I show genuine emotions with patients but with colleagues I suppress and fake more emotions. I’m in an assessment situation and I really want to appear good or better than I am. (Nursing Student 21)

The findings suggest that the program fostered deeper self-awareness through reflection, emotional honesty, and greater sensitivity toward others. Participants developed insight into their behaviors and values, enabling more authentic and confident engagement in clinical practice while also recognizing the ongoing tension between emotional genuineness and professional expectations [22, 55–57].

### Theme 3: development of emotional resilience

Emotional resilience was crucial since it enabled participants to effectively cope with and adapt to challenging or stressful situations in both clinical placement and life in general.

Academic work and performance used to stress me out a lot. I got really irritated and blamed myself a lot. Now I try to respond with calmness and handle stress better. I take a deep breath and reflect more about the situation. (Nursing Student 20)

I feel more prepared for practice now. It never used to be okay to refuse things at work but now I’ve learnt that it’s okay to say no if I’m not happy at work. Now I feel confident about saying no if I’m tired and I can express myself more instead of not saying anything. (Nursing Student 13)

While many participants felt being a student nurse on clinical placement was challenging, they grew stronger and more confident due to the genuine support they received from their peers.

I have some people there (group members/fellow students) who are really there for me now. It’s really lovely and it contributes to my feeling better in practice. Occasionally it can feel like we’re a bit in the way of what’s happening at the hospital, but now I feel it’s okay that I’m a student and I’m allowed to make mistakes and speak up about things I don’t fully understand. I’ve become more secure in my role out in practice. (Nursing Student 17)

The development I feel I’ve got from the group means I feel more present in meeting with patients. I feel calmer and less stressed, and more confident that I’ll become a good nurse. I’m good enough as I am and don’t need to get so stressed about not being able to get it right because I will get it right, as long as I believe in myself. (Nursing Student 4)

The participants also became more aware of the value of effective emotion regulation through reappraisal. This involved increasing their ability to change their mindset constructively:

Now I’m better at turning negative events into something positive. In my mind, what’s done is done, so I just have to move on. I try turning the negative emotion into something positive so that something good comes of it. (Nursing Student 1)

I had a situation at the hospital where I was responsible for a patient where I felt I was running in and out of the room all the time. At one point I got a bit tired of running in and out and never finishing, but then I just breathed for a bit and thought positively and crossed off everything I had got done on the list. (Nursing Student 11)

The findings suggest that the program contributed to the development of emotional resilience by helping participants manage stress, regulate emotions, and build confidence in clinical settings. This implies a growing ability to reframe challenges constructively and remain present, calm, and self-assured in demanding nursing roles [6, 14, 19].

## Discussion

Nursing students face challenges that can potentially lead to stress and impaired performance [1, 2]. The findings from this study highlight the value of a self-development program designed to enhance EI and mental well-being, which in turn improves nursing students’ ability to manage stress. The findings support the notion that enabling nursing students to consciously identify, express, and regulate their own emotions – and those of others (e.g. peers) can positively impact their stress management, professional awareness, and emotional development [6]. These findings align with previous EI interventions reporting improvements in emotional competencies, such as empathy, self-awareness, and emotional clarity among nursing students, aspects closely linked to mental well-being and effective stress management [52, 58–60]. However, our study differs from brief or skills-based EI interventions [61, 62] in that it emphasized ongoing personal reflection and peer dialogue, which appeared to foster deeper emotional insight. Additionally, our research suggests that teaching EI promotes the formation of a positive professional identity, emphasizing the importance of transformative learning activities that engage both cognitive and affective domains [63, 64]. This, in turn, nurtures appropriate attitudes, attributes, and professional values, fostering compassion [2]. This is partially supported by Teskereci et al. [65] even though no increase in EI level was found in their study. However, the authors stress the value of further exploring educational methods to develop EI in nursing students.

For the nursing students, fostering a sense of connectedness and psychological safety appeared to be important for enhancing their empathy and mental well-being. Social and relational emotions played a key role in helping students understand how people connect, increasing awareness of their environment and peers’ vulnerability, which is of significance for their future clinical role [66, 67]. Such experiences heightened a sense of connectedness, creating a greater feeling of meaningfulness, unity and joy, allowing the nursing students to feel safe, respected, and cared for ontologically. This sense of connection also helps counteract loneliness, which is an increasing challenge in nursing education [68, 69]. Further, it echoes the findings of Tabib et al. [52] who described a “ripple effect” of emotional intelligence training on midwife students’ sense of connectedness, self-awareness, and empowerment in practice. The study also highlighted how emotionally safe spaces supported transformation on both personal and professional levels. Developing reflexivity and cognitive reappraisal in nursing students better prepares them for the realities of nursing practice, contributing to sustained mental well-being and resilience [70–72]. In this context, cognitive reappraisal may foster a creative and adaptive interpretation of challenging situations [73, 74].

Nursing students also reported managing their emotions to suit specific contexts, using effective stress management strategies. In some cases, they masked or suppressed their feelings when lacking confidence, feeling unhappy, or striving to present a more professional demeanor. Notably, they often adopted this façade to align with the professionalism expectations of nursing (e.g. [75]). Through the self-development program, nursing students had the opportunity to become more aware of their own emotions and the emotions of others, recognizing their strategies for managing emotions contextually. In general, these strategies led to positive outcomes, enhancing their adaptability and mental well-being [52].

The findings from this study have implications for how nursing students are trained for the profession [67, 76, 77]. In addition to acquiring essential nursing knowledge and skills, it is equally important that students are trained in ‘soft’ skills [7, 39, 44, 70, 78]. These skills are critical for managing stress and coping with difficult situations, both during their education and in clinical placements [8, 10, 34]. Awareness of emotions and the ability to regulate and manage them also contribute to sound problem-solving and decision-making [54, 61, 72].

Incorporating an EI framework as a mechanism to build resilience and non-technical skills in nursing students is a promising approach for baccalaureate nursing education [5, 38, 79]. At the same time, nursing students must be supported in navigating the emotional tension between authenticity and professionalism, as emotional suppression or the faking of emotions – though common – may undermine mental well-being over time [57, 80].

Importantly, the group-based format in our study enabled students to connect with peers, share vulnerabilities, and build emotional resilience together. These findings highlight the value of integrating emotionally supportive and reflective spaces into nursing education. This allows students to process the demands of both academia and practice and to develop a sustainable, self-aware professional identity making space for empathy [52, 81, 82].

## Conclusion

This study provides evidence supporting the value of self-development programs within nursing education. The program created a psychologically safe environment where nursing students felt seen, supported and able to engage in open dialogue, fostering trust, self-reflection, and emotional growth. These experiences appear to contribute positively to students’ mental well-being and emotional competence. The findings underscore the relevance of integrating EI training in nursing education and peer feedback interventions. Such training can strengthen students’ abilities to recognize and regulate their emotions, navigate interpersonal challenges, and build resilience – skills essential for both academic success and the emotional demands of clinical practice. This supports the value of a stronger emphasis on structured EI and self-development training in nursing curricula, with the potential to inform educational strategies and policy aimed at promoting psychological readiness and long-term professional sustainability.

### Limitations

This qualitative study acknowledges certain limitations. While the findings offer valuable insights and contribute to hypothesis-generating theory and nuance, caution is advised when interpreting and drawing conclusions. The results are based on responses from two groups at a single university in Norway, which may limit their transferability. A potential limitation is the use of a self-selection strategy combined with a first come, first served approach, which may have introduced participation bias; students who chose to participate were likely more motivated or predisposed to personal development. Additionally, the impact of the COVID-19 pandemic may have influenced the outcomes. However, comprehensive reporting of the methods and the inclusion of participant quotes reinforce the dependability of the research. To minimize potential bias, an iterative process and regular discussions among co-authors fostered a multi-perspective approach, enhancing the overall trustworthiness of the study. Future research should explore the long-term effects of such interventions through longitudinal studies and implementation trials across diverse educational settings and student groups. This could strengthen transferability and deepen understanding of emotional development in nursing education.

## Electronic supplementary material

Below is the link to the electronic supplementary material.

Supplementary Material 1

## Data Availability

The datasets used and analysed during the current study are available fromthe corresponding author on reasonable request.
