# Supplementary material for: Enhancing emotional intelligence and mental well-being for stress management in nursing education – a qualitative study
Source: BMC Nurs. 2025 Jul 1;24:718. doi: 10.1186/s12912-025-03338-9 (PMC12211460; doi:10.1186/s12912-025-03338-9)
Supplement: Supplementary file 1 — Supplementary Material 1 [file 12912_2025_3338_MOESM1_ESM.docx]

**Supplementary file:** Questions in the interview guide (participants were asked to give examples and/or elaborate on each question)

| **Theme** | **Questions** |
| --- | --- |
| **Background information** | Age, gender, work experience from healthcare settings |
| **Mental well-being** | - How do you find your overall mental well-being as a student at this university? - Has your involvement in the self-development group contributed to your well-being and mental health? - Have you reflected on the extent to which participation in the self-development group has possibly influenced your academic achievements? - Could you characterize the community dynamic within the group? |
| **Self-Reflection and personal growth** | - In what ways, if any, do you believe that being part of the self-development group has contributed to your self-awareness and personal growth - In what ways, if any, has your understanding of your own emotions changed during the program? Can you share any examples? - In what way, if any, do you believe that your participation in the group has influenced how you use your emotions to perform academically or in clinical practice? - In what ways, if any, has your ability to understand and respond to the emotions of others been influenced by your participation in the group? - In what ways, if any, has participating in the self-development group been valuable, meaningful, or influenced your motivation? - In what ways, if any, do you think the skills or knowledge gained from the self-development group could be relevant to your future academic endeavors or clinical practice |

| **Emotion regulation/Stress** | - In what ways, if any, has participating in the group influenced your ability to regulate emotions, such as stress or frustration, in study situations or clinical practice? - Can you share examples of emotion regulation strategies you have used in educational settings/clinical practice? For instance, strategies like surface acting/faking, suppressing, or reappraising - In what ways, if any, has participating in the self-development group influenced your ability to manage stress? |
| --- | --- |

- Is there anything we haven’t talked about that you think should be included?
- Is there anything you’d like to add to what you’ve already told me?
